# Use of antiviral medications during pregnancy and the likelihood of preeclampsia in a population-based register study

**DOI:** 10.1038/s41598-025-09283-6

**Published:** 2025-07-01

**Authors:** Alice Zancanaro, Susanne Hesselman, Roxanne Hastie, Anna Fogdell-Hahn, Theodora Kunovac Kallak, Anna-Karin Wikström, Susanne Lager

**Affiliations:** 1 Department of Women’s and Children’s Health, Uppsala University, Uppsala, Sweden; 2 Center for Clinical Research, Falun, Sweden; 3 Department of Obstetrics and Gynecology, University of Melbourne, Heidelberg, Australia; 4 Affibody, Stockholm, Sweden

**Keywords:** Viral infection, Placenta, Pregnancy complications, Fetal growth restriction, Antivirals, Preeclampsia, Epidemiology, Pre-eclampsia, Viral infection

## Abstract

Viral infections and reactivations have been linked to several pregnancy complications. Antiviral medications are often prescribed as a prophylactic measure to prevent infant infection. This study examines associations between antiviral medication use during pregnancy and preeclampsia, thereby exploring links between viruses and such pregnancy complication. We analyzed data from 618,814 first-time mothers in Sweden (2007–2019) using four national registers. Of these 27,135 (4.4%) developed preeclampsia and 18,004 (2.9%) filled an antiviral medication prescription. Adjusted logistic regression with inverse probability of treatment weighting was used to evaluate associations between use of antiviral medication and preeclampsia. Antiviral medication use was associated with a reduced likelihood of preeclampsia (aOR 0.88; 95% CI, 0.81–0.96). Particularly, antiviral medication use was associated with a reduced likelihood of developing preeclampsia with delivery before 34 weeks gestation (aOR 0.66; 95% CI, 0.48–0.92) and preeclampsia with a small for gestational age infant (aOR, 0.74; 95% CI, 0.57–0.96). Timing of antiviral prescription filling was also investigated. Antiviral medication use in the third trimester was associated with a reduced likelihood of preeclampsia (aOR 0.77; 95% CI, 0.67–0.90), but not when used during the first two trimesters. Our study suggests that viral infections or reactivation might play a role in the etiology of preeclampsia, highlighting the need to further explore viral infections’ role in preeclampsia development.

**Supplementary Information:**

The online version contains supplementary material available at 10.1038/s41598-025-09283-6.

## Introduction

Preeclampsia is a pregnancy disorder characterized by high maternal blood pressure, coupled with organ dysfunction. As such, it is a significant contributor to global maternal morbidity. Preeclampsia poses a direct danger to mother and child, while also having a long-lasting effect on a woman’s life^1^. Treatment mainly focuses upon management of hypertensive symptoms, but the only remedy is delivery of the placenta. As a consequence, preeclampsia is one of the main causes for preterm birth^2^.

Preeclampsia can be classified as preterm (with delivery before gestational week 37) or term (with delivery after gestational week 37)^1,3^. Preterm preeclampsia is thought to be driven by abnormal placentation during early pregnancy^4,5^, while term preeclampsia is often characterized by a later decline in placental function^3^. The etiology of preeclampsia is multifactorial. Inflammation, endothelial dysfunction and the release of antiangiogenic factors by the placenta are recognized as contributing significantly to the development of the disease^6^. Recent studies have suggested that several viruses may increase the risk of developing preeclampsia. These include elevated levels of antibodies against rubella and herpes simplex virus 2 (HSV-2)^7^, the presence of human herpesvirus 6 in the feto-placental unit^8^, as well as maternal infection with SARS-CoV-2. However, conflicting findings regarding the association between SARS-CoV-2 infection and preeclampsia have been reported^9,10^. Infections with other ubiquitous viruses such as cytomegalovirus, Epstein-Barr virus and some herpesviruses are known risk factors during pregnancy and can lead to serious fetal outcomes, including miscarriage, congenital anomalies and permanent hearing and vision loss^11,12^. Among these pathogens, HSV-2, the primary cause of genital herpes and a prevalent infection in the general population, is of particular concern and a leading indication for antiviral prescription during pregnancy. Globally, 17.3% of live births occur among women with either prevalent or incident HSV-2 infection during pregnancy^13^. Without treatment, neonatal HSV-2 infection carries a high mortality rate, estimated at up to 60%^14,15^. Antiviral medications are often prescribed as a prophylactic measure during pregnancy to reduce viral shedding and risk of vertical transmission, and current clinical guidelines support their use to mitigate adverse neonatal outcomes^16,17^.

The aim of this population-based register study was to investigate the effects of viral infections and reactivations on preeclampsia development by examining the use of antiviral medications during pregnancy.

## Materials and methods

We undertook a register-based study using data from the Swedish Medical Birth Register (MBR), the Swedish National Patient Register (NPR), the Swedish National Prescribed Drug Register (NPDR) and Statistics Sweden. Since 1973, the Swedish MBR has been prospectively collecting information on all births in Sweden. This is facilitated by recording data from the mother´s first antenatal visit, until mother and child leave the hospital^18^. Data is usually recorded by midwives and physicians at clinical visits, then reported electronically by using a check-box system. Data on the mother´s medical history from in-patient care and specialist out-patient care is reported using *the International Statistical Classification of Diseases and Related Health Problems 10th revision* (ICD-10) codes collected from the NPR. Additionally, the NPDR collects information on all medications dispensed by Swedish pharmacies. We collected information on prescribed and filled prescriptions for antiviral medications and aspirin (routinely prescribed at low dosages for women at high risk of developing preeclampsia). Information on country of birth and years of education was collected from Statistics Sweden. Women were linked amongst registers through their personal identification number, which is assigned at birth or when achieving residency in Sweden. For all statistical analyses, we assumed compliance with the instructions for dispensed prescriptions. This study was approved by the Swedish Ethical Review Authority (approval number 2019–04925 with amendment number 2022 − 00922). According to Swedish legislation, informed consent from patients is not required for register-based studies. Access to data from these registers requires a request to the relevant government agency, following submission of a valid ethical review and approval. Consequently, in the context of our study, a statement on informed consent is not applicable, as Swedish law deems ethical approval to provide sufficient protection for participants in register-based research.

### Study population

Data was collected on all women giving birth to their first child in Sweden between 2007 and 2019. Pregnancies that ended in stillbirth after the 22nd gestational week were included. Twin pregnancies were excluded.

Gestational length at delivery was calculated based on a routine antenatal ultrasound for 91.4% of pregnancies, based on estimated gestational length after delivery for 4.3% of women, and based on last menstruation supported by estimated gestational length for 3.8% of women.

Maternal characteristics included: age at delivery, body mass index (BMI; calculated from height and weight at first antenatal visit), country of birth (classified as Sweden, other Nordic countries, North America and Europe, or other countries). Housing situation was defined as living with a partner, living in a single household, or other situation. Level of education was recorded as having less than 12 years of schooling (not completed high school), having completed high school, or having completed university. Information on smoking status, use of in vitro fertilization (IVF) and pre-gestational disorders (chronic hypertension, kidney disease, systemic lupus erythematosus, diabetes) was collected through MBR (using pre-defined checkboxes) and NPR (ICD-10 codes: pre-gestational diabetes: O240, O241).

Information on herpes simplex diagnosis during pregnancy (ICD-10 codes: A60, B00) was also collected as it is the primary reason for antiviral medication prescription during this period.

### Exposure

We defined exposure as filling an antiviral medication prescription, specifically for nucleosides and nucleotides, excluding reverse transcriptase inhibitors with *Anatomical Therapeutic Chemical* (ATC) classification code J05AB, during pregnancy or 90 days before estimated conception date. Antiviral medications prescribed in Sweden with ATC-code J05AB include: Aciclovir, Ganciclovir, Famciclovir, Valaciclovir, Cidofovir, Valganciclovir and Remdesivir. To investigate the significance of timepoint in antiviral medication use, women filling a prescription for antiviral medications were further divided into three categories based on when the first prescription was filled: before pregnancy (90 days before conception) or first trimester (up to week 12), second trimester (weeks 13 to 27), and third trimester (weeks 28 to delivery). The use of antiviral medications with ATC-code J05AB is considered safe during pregnancy^19–21^ and their use is not associated with an increase in birth defects^22^.

### Outcomes

Preeclampsia was the primary outcome, recorded as ICD-10 codes: O11, O14, O15 and O16 in MBR and NPR. A distinction was made between different preeclampsia outcomes, depending on gestational age at delivery or infant growth. The following analyses were repeated for each: preeclampsia with early preterm delivery defined as preeclampsia with delivery before week 34 (preeclampsia < 34 weeks), preterm preeclampsia defined as preeclampsia with delivery before week 37 (preeclampsia < 37 weeks), term preeclampsia defined as preeclampsia with delivery after week 37 (preeclampsia ≥ 37 weeks), and preeclampsia with a small-for-gestational-age infant (preeclampsia with SGA). SGA was defined as a birthweight below two standard deviations of mean birthweight for gestational age and fetal sex^23^.

### Statistical analysis

Population characteristics were presented according to antiviral medication use. Women taking antiviral medication were further described according to time point of first dispensed prescription. Results are presented as unadjusted and adjusted odds ratios, with a 95% confidence interval (OR or aOR with 95% CI).

As a form of sensitivity analysis, the group unexposed to antiviral medications was further divided according to HSV status. Differences in likelihood of developing preeclampsia between the three groups (I. Women unexposed to antivirals, HSV-, II. Women unexposed to antivirals, HSV + and III. Women exposed to antivirals) were explored using logistic regression.

All statistical analyses were performed using R Statistical Software (RStudio 2023.09.1 + 494 “Desert Sunflower”). P-values below 0.05 were considered statistically significant.

### Confounders

Possible confounders were evaluated by making a directed acyclic graph (DAG), considering clinical risk factor for preeclampsia from the existing literature (Supplementary Fig. 1). The confounders identified were: maternal age, BMI, country of birth, IVF use, smoking at first antenatal visit, pre-gestational diabetes, chronic hypertension, chronic kidney disease and systemic lupus erythematosus.

### Inverse probability of treatment weighting

The association between use of antiviral medications and the development of preeclampsia was explored using logistic regression with inverse probability of treatment weighting (IPTW). For this analysis, the control group included all women unexposed to antiviral medications. Briefly, IPTW is a method that can be used to adjust baseline characteristics of a population in an observational study^24,25^. IPTW was done in two steps. First, the propensity score for average treatment effect (ATE) was calculated using the exposure as the outcome and weights were stabilized with the *WeightIt* package^26^ in R. Covariate balance was assessed graphically using the *cobalt*^27^ R package. aOR with 95% CI were calculated using robust standard errors, computed with the R package *clubSandwich.*^28^ Three different imputations were made for missing BMI values and the analysis was repeated three times for each imputed BMI dataset. Imputations were made using the *mice*^29^ R package with predictive mean matching (pmm), using our confounders (as identified in the DAG) as predictors.

Model fit and aORs were calculated for each different BMI imputation, with results pooled together using Rubin´s combining rule^30^. For time of exposure subgroup analysis, a new propensity score was calculated for each of the three timepoints. Again, model fit and odds ratios were calculated for each different BMI imputation, with results pooled together.

For all analyses involving different preeclampsia outcomes, eight individuals were excluded because of unknown gestational age.

## Results

There were 618,867 women that gave birth to their first child between 2007 and 2019. Of these 53 filled their antiviral medication prescriptions after their delivery date and were excluded. In total, 618,814 women were included in this study.

Of the 618,814 primiparous women included, 18,004 (2.9%) used antiviral medications at some point during their pregnancy and 27,135 (4.4%) developed preeclampsia. Compared to women that were unexposed to antiviral medications, women prescribed antiviral medications were slightly older, had a lower BMI, were more likely to be born in Sweden, were more likely to have achieved higher education and less likely to smoke. Women taking antiviral medications also had slightly longer pregnancies and were less likely to give birth to a SGA infant (Table 1).

**Table 1:** Maternal characteristics of women included in the study giving birth to their first child in Sweden between 2007 and 2019.

	Overall	Unexposed to antivirals	Exposed to antivirals
N	618,814	600,810	18,004
Age	28.56 (5.05)	28.53 (5.05)	29.43 (5.06)
BMI, kg/m^2^	24.36 (4.51)	24.37 (4.53)	23.81 (4.03)
Missing	40,597	39,322	1,275
BMI			
<18.5	16,865 (2.9%)	16,439 (2.9%)	426 (2.5%)
18.5–24.9	360,454 (62.3%)	349,042 (62.2%)	11,412 (68.2%)
25.0–29.9	137,009 (23.7%)	133,448 (23.8%)	3,561 (21.3%)
30.0–34.9	45,066 (7.8%)	44,080 (7.9%)	986 (5.9%)
35.0–39.9	13,872 (2.4%)	13,608 (2.4%)	264 (1.6%)
≥40	4,951 (0.9%)	4,871 (0.9%)	80 (0.5%)
Missing	40,597	39,322	1,275
Country of birth			
Sweden	474,741 (76.8%)	459,031 (76.5%)	15,710 (87.4%)
Other Nordic	7,588 (1.2%)	7,355 (1.2%)	233 (1.3%)
North America and Europe	45,107 (7.3%)	44,298 (7.4%)	809 (4.5%)
Other	90,473 (14.6%)	89,246 (14.9%)	1,227 (6.8%)
Missing	905	880	25
Housing situation			
Living with partner	538,617 (91.3%)	523,083 (91.3%)	15,534 (90.9%)
Single	14,136 (2.4%)	13,657 (2.4%)	479 (2.8%)
Other situation	37,122 (6.3%)	36,037 (6.3%)	1,085 (6.3%)
Missing	28,939	28,033	906
Highest level of education			
Less than 12 years	82,761 (13.7%)	81,043 (13.9%)	1,718 (9.7%)
High school	160,399 (26.6%)	155,587 (26.6%)	4,812 (27.1%)
University	359,008 (59.6%)	347,809 (59.5%)	11,199 (63.2%)
Missing	16,646	16,371	275
Smoking	32,026 (5.2%)	31,256 (5.2%)	770 (4.3%)
In vitro fertilization	32,907 (5.3%)	31,901 (5.3%)	1,006 (5.6%)
Chronic hypertension	2,365 (0.4%)	2,298 (0.4%)	67 (0.4%)
Chronic kidney disease	2,767 (0.4%)	2,683 (0.4%)	84 (0.5%)
Diabetes (pre-gestational)	3,896 (0.6%)	3,774 (0.6%)	122 (0.7%)
Systemic lupus erythematosus	813 (0.1%)	782 (0.1%)	31 (0.2%)
Aspirin use	16,406 (2.7%)	15,875 (2.6%)	531 (2.9%)
Pregnancy length (days)	279 (14)	279 (14)	280 (13)
Missing	109	109	0
Preterm (delivery < 259 days)	35,998 (5.8%)	35,110 (5.8%)	898 (5%)
Birthweight (grams)	3,442 (567)	3,442 (568)	3,475 (533)
Missing	826	799	27
SGA	21,445 (3.5%)	20,929 (3.5%)	516 (2.9%)
Missing	1,026	998	28
LGA	11,736 (1.9%)	11,405 (1.9%)	331 (1.8%)
Missing	1,026	998	28

Data are presented as mean (standard deviation) or n (%). BMI, body mass index; LGA, large for gestational age; SGA, small for gestational age.

### Antiviral medications and preeclampsia

The sensitivity analysis showed that women unexposed to antivirals with a HSV diagnosis did not have an increased likelihood of developing preeclampsia. Their likelihood of developing preeclampsia was not significantly different from that of women unexposed to antivirals without a HSV diagnosis (Supplementary Table 1).

**Fig. 1:**
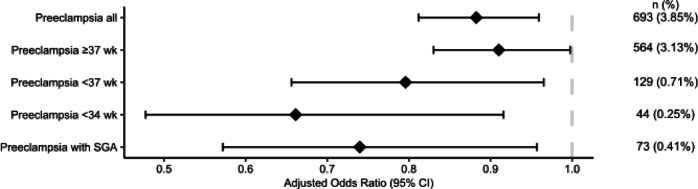
Forest plot showing adjusted odds ratios with 95% confidence intervals (CIs) for different preeclampsia outcomes according to antiviral use. Odds ratios were calculated using logistic regression adjusted with propensity scores and inverse probability of treatment weighting. Reference group: women unexposed to antivirals.

Women taking antiviral medications during pregnancy were less likely to develop preeclampsia than those who did not (3.8% vs. 4.4%, *P* < 0.001). IPTW was used to adjust for differences in baseline characteristics between groups. First, when adjusting for both baseline characteristics and confounding factors, exposure to antiviral medications was associated with a reduced likelihood of preeclampsia (aOR 0.88; 95% CI 0.81–0.96; *P* = 0.003). Next, the outcome of preeclampsia was divided into term (delivery *≥* 37 weeks gestation), preterm (delivery < 37 weeks gestation), early preterm (delivery < 34 weeks gestation) and preeclampsia with an SGA infant. Women exposed to antiviral medications had lower risks for developing all preeclampsia outcomes considered (Fig. 1).

### Timepoint of first exposure to antiviral medication and preeclampsia

Lastly, we investigated whether the timing of first exposure to antiviral medication was associated with preeclampsia. Exposure to antiviral medication starting before pregnancy and the first trimester was most common (54.6%; *n* = 9,822), followed by the third trimester (36.5%; *n* = 6,564). Fewer women started antiviral medication treatment during the second trimester (9.0%; *n* = 1,618). Supplementary Table 2 described our cohort according to time point of first prescription filling. Women exposed to antiviral medications for the first time during the third trimester had the lowest incidence of preeclampsia (3.9%), while women taking antiviral medications during the second trimester had the highest incidence (4.9%).

**Fig. 2:**
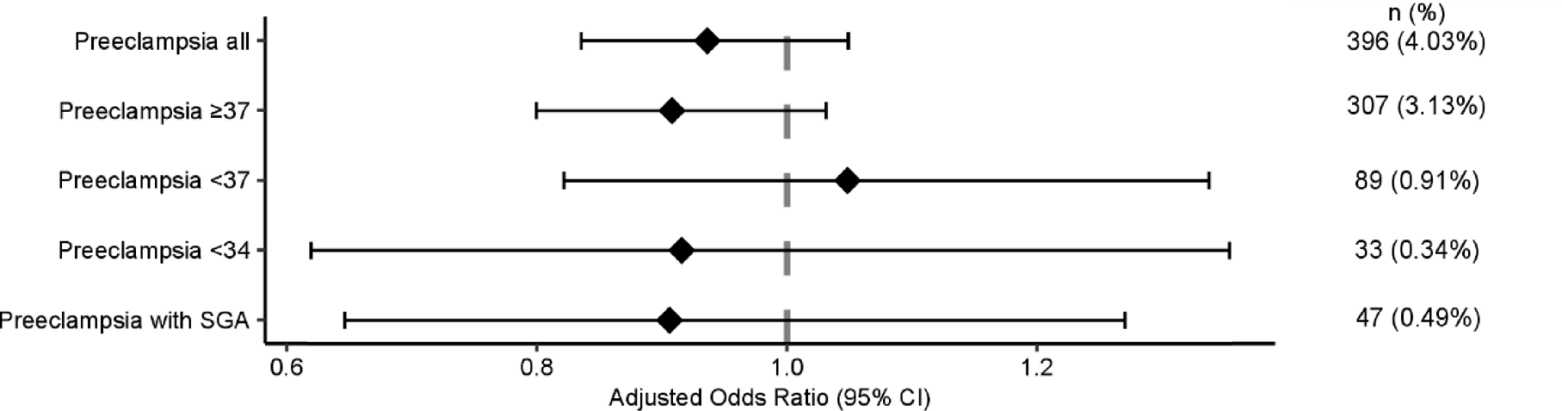
Forest plot showing adjusted odds ratios with 95% confidence intervals (CIs) for different preeclampsia outcomes according to antiviral use for women that started taking antivirals during **the first trimester** of pregnancy. Odds ratios were calculated using logistic regression adjusted with propensity scores and inverse probability of treatment weighting. Reference group: women unexposed to antivirals.

**Fig. 3:**
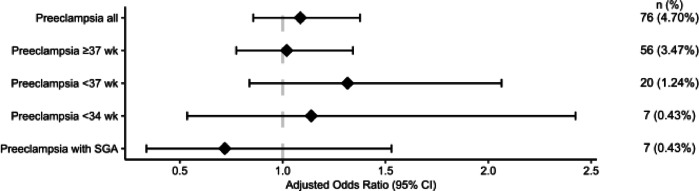
Forest plot showing adjusted odds ratios with 95% confidence intervals (CIs) for different preeclampsia outcomes according to antiviral use for women that started taking antivirals during **the second trimester** of pregnancy. Odds ratios were calculated using logistic regression adjusted with propensity scores and inverse probability of treatment weighting. Reference group: women unexposed to antivirals.

**Fig. 4:**
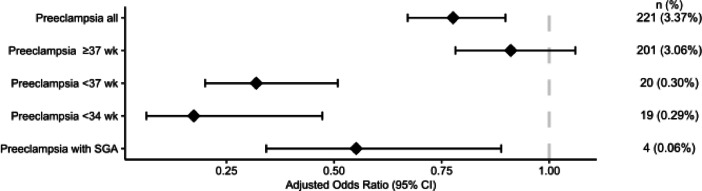
Forest plot showing adjusted odds ratios with with 95% confidence intervals (CIs) for different preeclampsia outcomes according to antiviral use for women that started taking antivirals during **the third trimester** of pregnancy. Odds ratios were calculated using logistic regression adjusted with propensity scores and inverse probability of treatment weighting. Reference group: women unexposed to antivirals.

When adjusting for both baseline characteristics and possible confounders, exposure to antiviral medications during the beginning of pregnancy (Fig. 2), or during the second trimester (Fig. 3), did not alter the likelihood of developing preeclampsia. Exposure to antiviral medication commenced during the third trimester was associated with a lower likelihood for developing preeclampsia (aOR 0.77; 95% CI 0.67–0.90; *P* < 0.001) (Fig. 4).

## Discussion

In this cohort of 618,814 women giving birth to their first child in Sweden, we found that use of antiviral medications during pregnancy was associated with a reduced likelihood of developing preeclampsia. This association was found for all preeclampsia outcomes considered. Preeclampsia with delivery before 34 weeks gestation and preeclampsia with birth of an SGA infant, showed the largest decreases in likelihood. Interestingly, we found that women prescribed antiviral medications were less likely to have SGA infants and more likely to have longer pregnancies, when compared to women unexposed to antiviral medications. When repeating the analysis according to timepoint of first antiviral medication prescription filling, a significant reduction was only found in women filling their first prescription during the third trimester.

Several viruses have been implicated, to varying degrees, in the development of preeclampsia^31^. While viral infections may justify the use of an antiviral prescription during pregnancy, the infection itself may not be the direct cause of preeclampsia. For instance, several members of the *Herpesviridae* family cause lifelong persistent infection through latency. These viruses may reactivate. Various stressors can lead to viral reactivation, of which co-infection with other pathogens is such a stressor^32^. In these cases, the antiviral medication may not only have helped with the symptomatic viral infection, but also prevented consequent reactivation of other viruses, potentially mitigating systemic inflammation or immune dysregulation that could contribute to preeclampsia. Similarly, some opportunistic viruses may also reactivate once a bacterial infection has compromised the immune system. It has been reported that women using antibiotic medication, as well as women with urinary tract infections during pregnancy, have a higher likelihood of developing preeclampsia^33^.

As for what happens during a viral infection and how antiviral medications could help lower the likelihood of developing preeclampsia, especially during the third trimester, we can only speculate. Although different viruses will have different routes of infection, it can generally be said that a viral infection will trigger an activation of the immune system^34–36^. It is possible that an additional increase in maternal inflammation (especially during the third trimester, when inflammation levels are naturally heightened)^37^ due to viral infection could contribute to a pathological increase, leading to preeclampsia. Antiviral medications may help mitigate these risks by reducing viral load, thereby dampening the immune activation that leads to both systemic and localized placental inflammation.

One limitation of our study is associated with assumptions that women who filled a prescription for antiviral medications actually used them as intended. We lack information concerning dosage, treatment duration, and clinical indication. While we do not have access to specific medication names, the prescriptions fall under ATC-code J05AB, which according to publicly available data from the Swedish National Board of Social Affairs and Health, primary includes Acyclovir and Valaciclovir, accounting for 99.5% of dispensed prescription in this category^38^. We are also limited by the fact that not all individuals having a viral infection are prescribed antiviral medications which may lead to exposure misclassification. Additionally, women who deliver preterm or very preterm may not have developed preeclampsia simply because the pregnancy ended before the condition could manifest. It is also important to consider that women who begin antiviral treatment in the third trimester have sustained their pregnancies to that point, which may indicate a healthier cohort. This could introduce selection bias and overestimate the protective association observed. From our registers, we are also missing the date of preeclampsia diagnosis. Although women generally are diagnosed with preeclampsia later in their pregnancy, it is possible that some had already been diagnosed with preeclampsia when filling their antiviral medication prescription.

The main strength of this study was use of the extensive Swedish nationwide registers, which allowed us to include a large cohort with large amounts of data without relying on self-reported information and thereby not being subject to recall bias. We were also not subject to selection bias, as almost all women giving birth to their first child in Sweden were included. The observed incidence of preeclampsia in our cohort is consistent with global estimates of between 3 and 5%, which supports the representativeness of our population. The percentage of pregnant women being prescribed antiviral medications also corresponds well with previous reports of antiviral use in Nordic countries^22^. The differences in numbers of prescription fillings at different timepoints during pregnancy was expected as, in Sweden, women are prescribed antivirals against recurrent HSV infection either at the beginning of pregnancy or 10 days before delivery as a prophylactic measure. The sensitivity analysis did not show any differences between women unexposed to antivirals despite differences in HSV status, further strengthening our hypothesis that women taking antivirals have a lower likelihood of developing preeclampsia compared to the unexposed population.

## Conclusion

Our findings suggest that antiviral medications may have a preventive effect on the development of preeclampsia, indicating that viral infections may be a contributing factor in development of the disease. From our data, we can only speculate what mechanisms underly these results. Therefore, further pre-clinical and clinical studies concerning the effect of viral infections and the use of antiviral medications during pregnancy are warranted.

## Electronic supplementary material

Below is the link to the electronic supplementary material.

Supplementary Material 1

## Data Availability

This study is produced under Swedish law where register data is made available to researchers upon request to a government agency if approved by an ethics committee. The data that supports the findings of this study are available from the Swedish National Board of Health and Welfare upon request. The exact instructions used to extract the data from the registers can be made available by the corresponding author upon reasonable request.
