# Supplementary material for: Use of antiviral medications during pregnancy and the likelihood of preeclampsia in a population-based register study
Source: Sci Rep. 2025 Jul 1;15:20809. doi: 10.1038/s41598-025-09283-6 (PMC12214669; doi:10.1038/s41598-025-09283-6)
Supplement: Supplementary file 1 — Supplementary Material 1 [file 41598_2025_9283_MOESM1_ESM.pdf]

Use of antiviral medications during pregnancy and the likelihood of preeclampsia in a population-based register study

Supplementary tables and figures

Supplementary table 1. Crude and adjusted ORs of developing preeclampsia for women exposed to antivirals and women unexposed to antivirals with a HSV diagnosis.

	Unexposed to antivirals, HSV– (n=586,919) Reference	Unexposed to antivirals, HSV+ N= 13,891			Antiviral use N=18,004		
Outcome	**N (%)**	**N (%)**	**Odds ratio (95% CI)**		**N (%)**	**Odds Ratio (95% CI)**	
			**Crude**	**Adjusted[a]**		**Crude**	**Adjusted[a]**
Preeclampsia	25,813 (4.4%)	629 (4.5%)	1.03 (0.95 – 1.12)	0.99 (0.91 – 1.08)	693 (3.8%)	0.87 (0.81 – 0.94)	0.89 (0.81 – 0.97)

[a]ORs adjusted for maternal age, BMI, country of birth, in vitro fertilization use, smoking at first antenatal visit, pre-gestational diabetes, chronic hypertension, chronic kidney disease and systemic lupus erythematosus. CI; confidence interval.

Supplementary table 2. Characteristic of women taking antiviral medications according to first timepoint of prescription filling.

Characteristic	Before pregnancy - First trimester	Second trimester	Third trimester	Unexposed
**N**	**9,822**	**1,618**	**6,564**	**600,810**
Age	29.35 (5.09)	28.92 (5.30)	29.66 (4.94)	28.53 (5.05)
BMI, kg/m^2	23.80 (4.01)	24.06 (4.18)	23.76 (4.00)	24.37 (4.53)
Missing	705	110	460	39,322
**BMI**				
<18.5	219 (2.4%)	44 (2.9%)	163 (2.7%)	16,439 (2.9%)
18.5 – 24.9	6,248 (68.5%)	990 (65.6%)	4,174 (68.4%)	349,042 (62.2%)
25 – 29.9	1,912 (21%)	338 (22.4%)	1,311 (21.5%)	133,448 (23.8%)
30.0 – 34.9	557 (6.1%)	96 (6.4%)	333 (5.5%)	44,080 (7.9%)
35.0 – 39.9	140 (1.5%)	31 (2.1%)	93 (1.5%)	13,608 (2.4%)
≥ 40	41 (0.4%)	9 (0.6%)	30 (0.5%)	4,871 (0.9%)
Missing	705	110	460	39,322
**Country of birth**				
Sweden	8,739 (89.1%)	1,341 (83%)	5,630 (86%)	459,031 (76.5%)
Other Nordic	111 (1.1%)	19 (1.2%)	103 (1.6%)	7,355 (1.2%)
North America and Europe	383 (3.9%)	95 (5.9%)	331 (5.0%)	44,298 (7.4%)
Other	575 (5.9%)	161 (10.0%)	491 (7.5%)	89,246 (14.9%)
Missing	14	2	9	880
**Housing situation**				
Living with partner	8,439 (90.5%)	1,378 (89.4%)	5,717(91.7%)	523,083 (91.3%)
Single	276 (3.0%)	42 (2.7%)	161 (2.6%)	13,657 (2.4%)
Other situation	609 (6.5%)	121 (7.9%)	355 (5.7%)	36,037 (6.3%)
Missing	498	77	332	28,033
**Highest level of education**				
Less than 12 years	903 (9.3%)	227 (14.3%)	588 (9.1%)	81,043 (13.9%)
High School	2,715 (28.0%)	439 (27.6%)	1,658 (25.8%)	155,587 (26.6%)
University	6,083 (62.7%)	925 (58.1%)	4,191 (65.1%)	347,809 (59.5%)
Missing	121	27	127	16,371

Characteristic	Before pregnancy - First trimester	Second trimester	Third trimester	Unexposed
**N**	**9,822**	**1,618**	**6,564**	**600,810**
Smoking	430 (4.4%)	80 (4.9%)	260 (4.0%)	31,256 (5.2%)
In vitro fertilization	497 (5.1%)	97 (6.0%)	412 (6.3%)	31,901 (5.3%)
Chronic hypertension	32 (0.3%)	10 (0.6%)	25 (0.4%)	2,298 (0.4%)
Chronic kidney disease	42 (0.4%)	10 (0.6%)	29 (0.4%)	2,683 (0.4%)
Diabetes (pre-gestational)	84 (0.9%)	9 (0.6%)	29 (0.4%)	3,774 (0.6%)
Systemic lupus erythematosus	18 (0.2%)	5 (0.3%)	8 (0.1%)	782 (0.1%)
Aspirin use	315 (3.2%)	53 (3.3%)	163 (2.5%)	15,875 (2.6%)
Pregnancy length (days)	279 (14)	278 (15)	282 (10)	279 (14)
Missing	0	0	0	109
Birthweight (grams)	3,444 (550)	3,433 (562)	3,533 (493)	3,442 (569)
Preterm (delivery <259 days)	617 (6.28%)	112 (6.92%)	169 (2.6%)	35100 (5.8%)
SGA	285 (2.9%)	51 (3.2%)	180 (2.7%)	20,929 (3.5%)
Missing	17	3	8	998
LGA	178 (1.8%)	47 (2.9%)	106 (1.6%)	11,405 (1.9%)
Missing	17	3	8	998

Data are presented as mean (standard deviation) or n (%). BMI, body mass index; LGA, large for gestational age; SGA, small for gestational age.

Supplementary figure 1

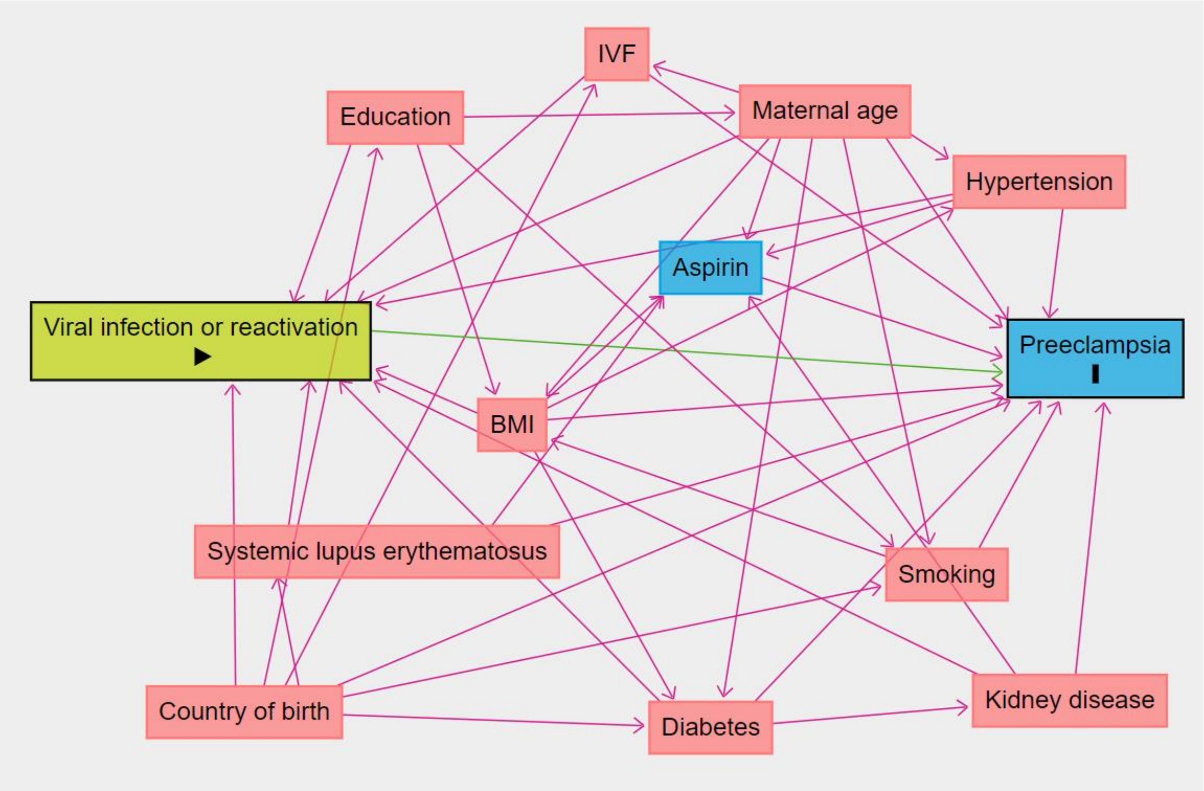

Supplementary figure 1. DAG illustrating our potential confounders in pink, our exposure in green and our outcome in blue. All directed arrows denote a potential confounding effect.
